# Comparison of diode laser and sonic activation in the removal of root canal filling material from curved canals: an in-vitro study

**DOI:** 10.1186/s12903-026-08807-4

**Published:** 2026-06-01

**Authors:** Zübeyde Gökçe Ürün, Barış Ürün, Özgür Tosun

**Affiliations:** 1 Faculty of Dentistry, Department of Endodontics, Niğde Ömer Halisdemir University, Niğde, Türkiye; 2 Faculty of Medicine, Department of Biostatistics, Near East University, TRNC, Mersin 10, Nicosia, Türkiye

**Keywords:** Curved canals, Eddy, Diode- laser, Retreatment

## Abstract

**Background:**

Retreatment in curved root canals, particularly achieving complete canal cleanliness, remains a clinical challenge and an ongoing subject of investigation. This study evaluated the efficiency of sonically activated irrigation (EDDY) and diode laser–activated irrigation (DL) in removing filling material from simulated curved canals.

**Methods:**

Thirty-three J-shaped resin blocks were instrumented to 25/06 apical size and obturated with single-cone resin sealer and gutta-percha. Retreatment was performed using ProTaper Universal Retreatment files. Samples were allocated to needle irrigation (NI, control), EDDY, or diode laser (DL) groups. NI and EDDY protocols were applied for 3 × 20 s, while the DL protocol consisted of four 5-second activation cycles. Sections at apical (2 mm), middle (6 mm), and coronal (10 mm) levels were examined at ×40 magnification. ImageJ, image analysis software was used to calculate the percentage of remaining obturation material. Two-way repeated measures ANOVA was used (*p* ≤ 0.05).

**Results:**

EDDY achieved significantly superior cleaning at the apical 2 mm and middle 6 mm levels compared with both NI and DL (*p* < 0.001). DL showed no significantly better cleaning efficiency than NI at any level (*p* > 0.05). In both NI and DL groups, filling material removal effectiveness differed significantly among the apical, middle, and coronal thirds (*p* < 0.05). In the coronal third, no statistically significant differences were observed among all groups (*p* > 0.05).

**Conclusion:**

Although none of the methods achieved the complete removal of filling materials, EDDY was significantly more effective than needle irrigation and diode laser activation in the apical and middle region of curved canals. These findings suggest that, to significantly improve apical cleanliness, clinicians should consider sonic activation during retreatment in curved canals.

## Background

Primary root canal treatment may fail due to several causes, such as coronal or apical leakage, inadequate canal cleaning, or poor filling quality. Non-surgical root canal retreatment may be preferred after unsuccessful endodontic treatments, as it provides the opportunity to clean the intra-root infection source once more [1]. Complete removal of the existing root canal filling, disinfection of the canal, and obturation are essential for treatment success to regain access to healthy periapical tissues [2]. Root canal filling residues that cannot be completely removed from the root canal walls may lead to the harboring of microorganisms, which will negatively affect treatment success [1]. However, studies have shown that old gutta-percha and root canal fillings cannot be completely removed, especially in canals with complex root canal anatomy [3–5].

This problem is addressed through the use of irrigation activation procedures. The sonic irrigation device EDDY (VDW, Munich, Germany) utilizes an air scaler to generate sonic vibrations at frequencies of up to 6000 Hz. These vibrations are transmitted to a flexible polyamide tip, producing a high-amplitude, three-dimensional oscillating motion. Such motion induces both cavitation and acoustic streaming—mechanisms that enhance the penetration and efficacy of irrigants within the complex root canal anatomy. In contrast to conventional ultrasonic activation systems, EDDY’s polymer-based tip and sonic-driven mechanism facilitate effective irrigant agitation without direct contact with canal walls, thereby reducing the risk of dentinal damage while improving irrigant distribution [6–8].

Diode lasers have emerged as valuable tools in endodontic therapy, particularly for enhancing root canal disinfection. Their irradiation has been shown to not only improve antimicrobial efficacy but also induce morphological changes in dentin that occlude dentinal tubules, thereby enhancing the sealing of the root canal system [9]. While diode lasers are generally considered less effective than Er: YAG lasers due to their lower absorption in water-based irrigants, they remain popular due to their cost-effectiveness and availability [10]. Studies have also reported that diode lasers can facilitate the removal of root canal filling materials such as gutta-percha and bioceramic sealers during retreatment procedures [10, 11].

Due to its flexible polyamide tip, the EDDY system is expected to advance more effectively to the working length, particularly in curved root canals [12], and diode lasers have been shown to enhance retreatment outcomes; however, to the best of our knowledge, no studies have specifically investigated the efficacy of either method in the apical third of curved canals. Therefore, the present study aims to evaluate the efficacy of EDDY and diode laser activation procedures in removing filling material remnants during the final irrigation protocol following retreatment in curved, transparent resin blocks.

The null hypothesis of this study is that both activation methods are equally effective, and there will be no difference between groups and root canal levels.

## Materials and methods

### Sample size calculation

The sample size of *n* = 11 per group was determined based on effect sizes reported in published in vitro retreatment studies employing comparable three-group designs. Using G*Power 3.1, with a Cohen’s f of 0.55 (derived from a mean group difference of approximately 15% points and a pooled SD of approximately 10%, consistent with prior literature), a significance level of α = 0.05, and a desired power of 0.80, the minimum required sample size was estimated at *n* = 9 per group. The present study used *n* = 11 per group, providing sufficient power for the primary between-group comparisons [13].

### Root canal model

In this study, a total of 33 J-shaped Endo Training Resin Blocks (ETRB; Dentsply Maillefer, Ballaigues, Switzerland), each with a standardized curvature of 40°, were used to simulate curved root canals.

### Canal preparation and obturation

The apical patency of each canal was confirmed using a size #10 K-file (Dentsply Maillefer), and the working length was determined. All samples were prepared to size 25/06 using the T-Endo MUST file system (Dentac, Istanbul, Turkey) by a single experienced operator. Each file was used only once during the preparation procedure. During instrumentation, the canals were irrigated with 5.25% sodium hypochlorite (NaOCl). The canals were then obturated using the matched single cone technique with an epoxy resin-based root canal sealer (AH Plus, Dentsply Sirona, North Carolina, US) After root canal obturation, all samples were stored in an incubator at 37 °C and 100% relative humidity for 14 days to allow sealer setting.

### Retreatment procedures

All samples underwent retreatment procedures using the ProTaper Retreatment^®^ system (Dentsply Maillefer, Ballaigues, Switzerland), following the manufacturer’s instructions. The D1 (30/09), D2 (25/08), and D3 (20/07) files were used sequentially, with 5.25% sodium hypochlorite (NaOCl) irrigation applied between files. After using retreatment files, the apical diameter was enlarged to size 30, one size larger than that of the initial root canal treatment, to replicate the clinical scenario and improve apical cleaning as well as the removal of previous materials. All procedures were performed under a dental operating microscope (Leica Microsystems GmbH, Wetzlar, Germany) at 40× magnification.

### Irrigation procedures

All samples were randomly divided into three groups according to the final irrigation protocol (*n* = 11). A 5.25% NaOCl solution was used for irrigation activation. Needle irrigation (NI) with a 30G side-vented irrigation needle (Scope Endo, Yozgat, Turkey) served as the control group. Irrigation was performed for 20 s using a 30-gauge side-vented needle inserted 2 mm short of the working length, with the needle tip moved back and forth during the procedure. The process was repeated three times, with the irrigation solution refreshed each time. Subsequently, the canals were rinsed with 2.5 mL of distilled water, and the same irrigation procedure was repeated using 5 mL of 17% EDTA solution.

In the EDDY group, the canal was filled with 5.25% NaOCl, and the EDDY was placed 1 mm short of the working length. It was activated at 6000 Hz with a 4 mm back-and-forth movement, according to the manufacturer’s recommendations. The protocol was completed by repeating the procedure three times, each lasting 20 s. Subsequently, the canals were rinsed with 2.5 mL of distilled water, and the same irrigation procedure was repeated using 5 mL of 17% EDTA solution.

In Diode Laser (DL) group, 5.25% NaOCl was introduced into the canal and activation was performed using a 980 nm diode laser (LazonLaser, Lazon Medical Tech Co., Xi’an, China) with a 200 μm initiated fiber tip. The laser was operated in pulsed mode (1.5 W peak power, 50% duty cycle). The fiber tip was positioned 1 mm short of the working length and activated. It was then withdrawn in a spiral motion at a constant speed of approximately 2 mm/s. The irrigation protocol consisted of four cycles, with each cycle involving 5 s of laser activation followed by a 20-second pause, resulting in a total irradiation time of 20 s per canal. Total delivered energy of approximately 15 Joules per canal (Average power 0.75 W × 20 s). Subsequently, the canals were rinsed with 2.5 mL of distilled water, and the same irrigation procedure was repeated using 5 mL of 17% EDTA solution.

All samples were finally irrigated with 5 mL of saline and dried using paper points.

### Images and image analysis

**Fig. 1:**
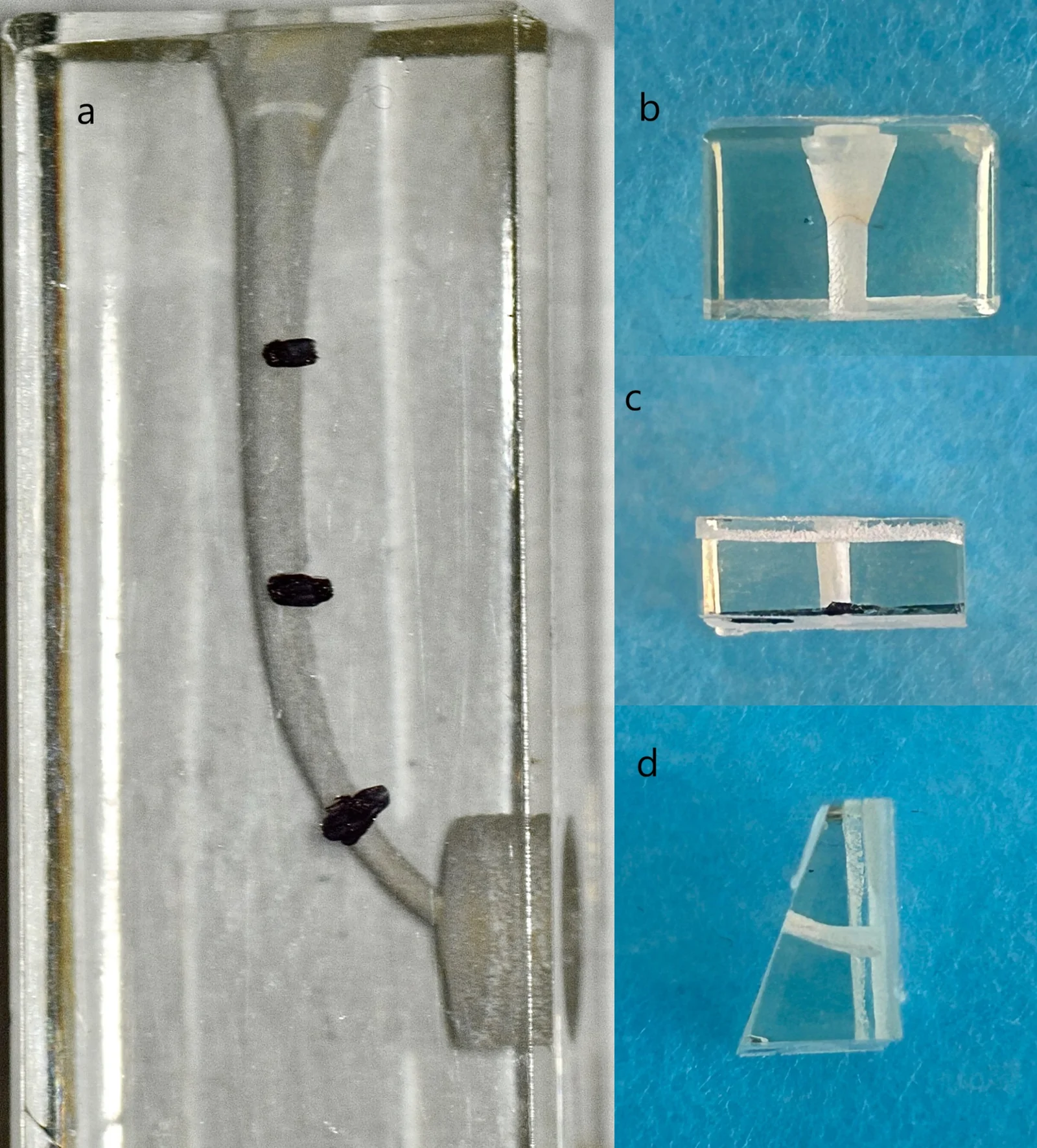
Sections of the canal at 2, 6, and 10 mm from the apex (apical, middle, and coronal thirds). (**a**) Marking of the section levels prior to the sectioning procedure, (**b**) coronal level (10 mm), (**c**) middle level (6 mm), (**d**) apical level (2 mm)

**Fig. 2:**
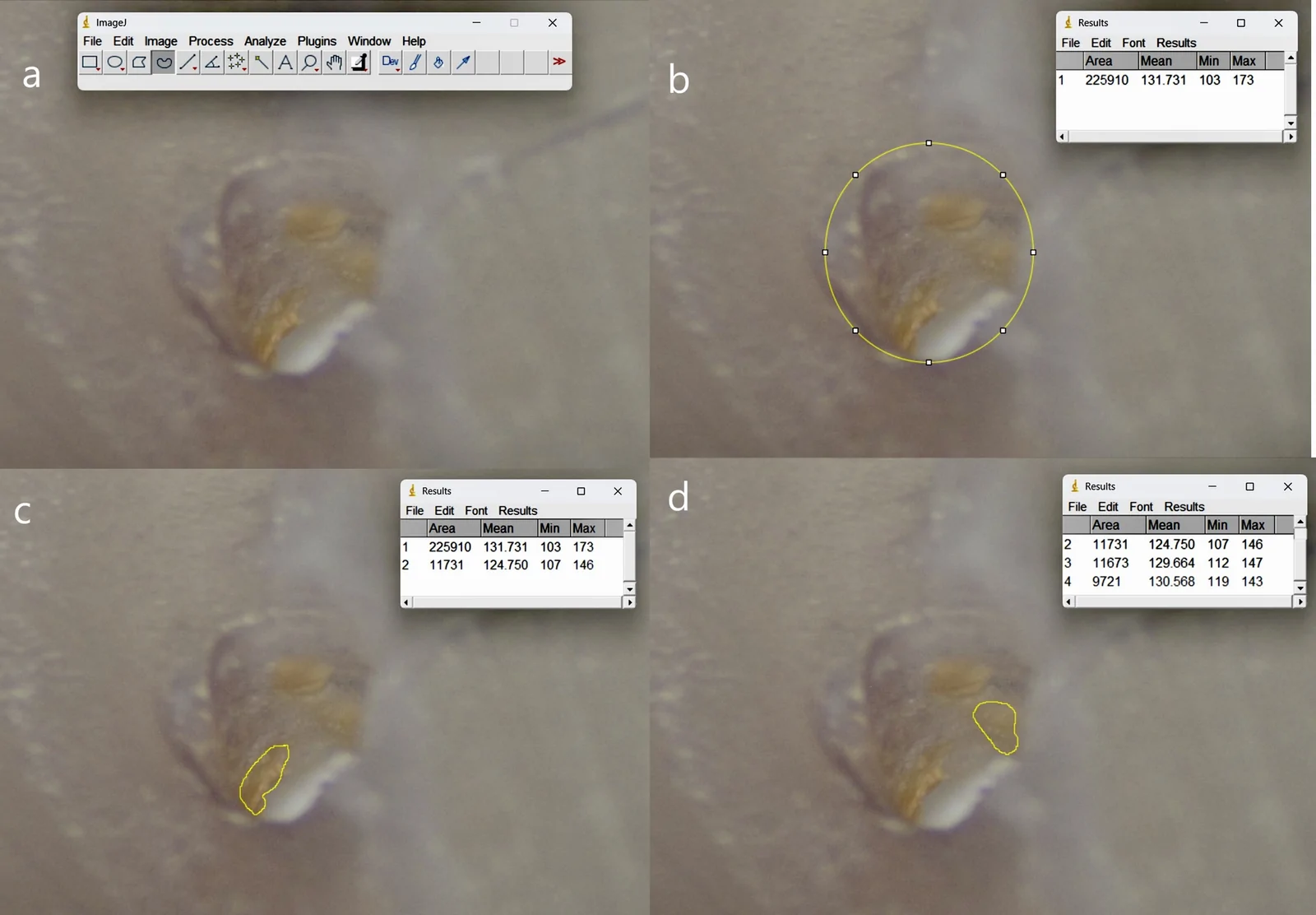
Representative sequence of image analysis using ImageJ. (**a**) raw image, (**b**) measurement of total canal area, (**c**, **d**) tracing and measurement of residual filling materials

The samples were sectioned perpendicular to the long axis of the canal at three pre-specified levels: at 2 mm (apical third), 6 mm (middle third), and 10 mm (coronal third) from the working length, using a diamond wafering blade with a thickness of 0.3 mm (Fig. 1). The obtained sections were photographed at 40× magnification using a digital camera (D5000, Nikon, Tokyo, Japan) integrated into an operating microscope. All images were captured under identical microscope and camera settings, ensuring consistency in pixel-based measurements across all specimens. Each segment was captured from three standardized angles by rotating the sample 120° between images, thereby assuring comprehensive visualization of the canal’s circumference. All images were transferred to ImageJ 1.38x image analysis software (National Institutes of Health, Bethesda, MD, USA) and analysis was performed through a four-step sequence (Fig. 2):

1. Digital micrograph was imported into the software;
2. The root canal area to be examined was circumscribed in the photograph to establish the region of interest.
3. The areas occupied by residual filling materials (gutta-percha and sealer) were manually traced and measured;
4. All measurements were recorded in pixels.

For each section, measurements obtained from the three standardized images were averaged, and the percentage of remaining obturation material was calculated by dividing the residual material area by the total canal area and multiplying by 100. Image analysis was performed on coded images by a single examiner who was blinded to group allocation at the time of measurement.

### Statistical analysis

Prior to inferential testing, the Shapiro-Wilk test was applied to assess normality of data distribution within each group at each canal level. The normality assumption was met for the majority of cells; where marginal departures were identified, normality of pairwise differences - the assumption of primary relevance in a repeated measures design - was confirmed. Mauchly’s test of sphericity was applied to the within-subjects factor; the sphericity assumption was violated, and the Greenhouse-Geisser correction was therefore applied to all within-subjects effects. A two-way mixed-design ANOVA was conducted, with irrigation method (DL, EDDY, NI) as the between-subjects factor and canal measurement level (2 mm, 6 mm, 10 mm) as the within-subjects factor. Post-hoc pairwise comparisons for both within-group and between-group effects were performed using Tukey’s HSD test. Effect size was reported as partial eta squared (η²p). P-values are reported as exact values throughout; where *p* < 0.001, this notation is used in accordance with standard reporting guidelines (APA, 7th ed.; CONSORT), as reporting further decimal places would imply a level of precision beyond what is statistically meaningful. All statistical analyses were performed using GraphPad Prism (Demo Version 10.3.1 (464) for Mac, GraphPad Software, San Diego, CA, USA).

## Results

The amounts of residual filling material at different canal levels in the EDDY, DL, and NI groups are presented in Tables 1 and 2. Two-way ANOVA revealed significant main effects of irrigation group (F [2,30] = 25.31, *p* < 0.001, η²*p* = 0.628) and canal level (F[2,60] = 60.85, *p* < 0.001, η²*p* = 0.670), as well as a significant interaction between group and canal level (F[4,60] = 5.86, *p* < 0.001, η²*p* = 0.281).

**Table 1:** Means ± SD of remaining filling material (expressed as percentage) at 2, 6 and 10 mm from the WL

		Descriptive Statistics			RM-ANOVA (GG-corrected)			
Variable	Canal Level	DL (*n* = 11) Mean ± SD	EDDY (*n* = 11) Mean ± SD	NI (*n* = 11) Mean ± SD	Effect	F (df)	*p*	η²*p*
	2 mm (Apical)	30.78 ± 16.56	13.31 ± 9.05	38.59 ± 6.11	Group	F(2; 30.0) = 25.31	**< 0.001**	0.628
Residual Filling Material (%)	6 mm (Middle)	13.32 ± 7.58	5.41 ± 3.85	18.77 ± 7.99	Canal Level	F(2; 60.0) = 60.85	**< 0.001**	0.670
	10 mm (Coronal)	4.32 ± 3.65	5.35 ± 4.07	8.58 ± 5.30	Group x Canal Level	F(4; 60.0) = 5.86	**< 0.001**	0.281

RM-ANOVA: Repeated measures ANOVA (Type III), Greenhouse-Geisser corrected. η²p: Partial eta-squared (small:0.01 - <0.06; medium: 0.06–0.14; large: >0.14). *DL* Diode Laser, *NI* Needle Irrigation

The *p*-values ​​indicated in bold are statistically significant

Within-group comparisons revealed that residual material decreased significantly across canal levels in the DL group and in the NI group (Table 2)*.* In the EDDY group, only the 2 mm vs. 6 mm comparison reached significance (*p* = 0.016), indicating homogeneous cleaning efficacy across the middle and coronal thirds with EDDY (Table 2).

Between-group comparisons showed that EDDY achieved significantly lower residual filling material than both DL (*p* = 0.019) and NI (*p* < 0.001) at the apical 2 mm level, and than both DL (*p* = 0.020) and NI (*p* < 0.001) at the middle 6 mm level. No significant between-group differences were observed at the coronal 10 mm level. DL did not differ significantly from NI at any canal level (Table 2)*.*

**Table 2:** Statistical comparisons of canal levels within each irrigation activation method (*p*-values)

		Within-Group (Tukey HSD)				Between-Group (Tukey HSD)		
Variable	Group	2–6 mm	2–10 mm	6–10 mm	Canal Level	DL–EDDY	DL–NI	EDDY–NI
	DL	**0.012**	**0.002**	**0.020**	2 mm (Apical)	**0.019**	0.338	**< 0.001**
Residual Filling Material (%)	EDDY	**0.016**	0.086	0.999	6 mm (Middle)	**0.020**	0.252	**< 0.001**
	NI	**< 0.001**	**< 0.001**	**0.032**	10 mm (Coronal)	0.808	0.100	0.270

*DL* Diode Laser, *NI* Needle Irrigation

The *p*-values ​​indicated in bold are statistically significant

## Discussion

Successful endodontic retreatment relies on the complete removal of previous filling materials and the effective elimination of residual infected tissues that may cause treatment failure. This challenge becomes even more pronounced in curved root canals, where the removal of residual filling materials from the curved regions is particularly difficult during retreatment procedures. To overcome these challenges, the use of irrigation activation techniques has been widely investigated in several studies [4, 6, 13, 14].

In this study, the matched-taper single gutta-percha cone approach was used for root canal obturation. Previous studies have reported that tapered cones are well adapted to the geometry of the prepared root canal [15, 16]. This method is considered a comparable option to other obturation techniques because it has been shown to effectively obturate narrow and curved canals and is widely regarded [16, 17].

Because resin-based sealers adhere to root canal dentin better than conventional zinc oxide eugenol or glass ionomer sealers, they have become more popular [18]. Epoxy resin-based sealers, such AH Plus can generate covalent connections without the need for dentine surface treatment. According to reports, AH Plus’s binding strength to dentine is even stronger than that of dual-cure resin-based sealers [19]. For these reasons, AH Plus sealer was preferred in the present study.

Through its multidirectional motion, EDDY induces acoustic streaming and cavitation, enhancing irrigant activation [20]. In their study using single-rooted straight canals, Çıkrık et al., found that the EDDY group performed significantly better than the conventional needle irrigation group at all three canal levels following retreatment [13]. Similarly, in our study, EDDY demonstrated better results than the control group at all levels; however, this difference was not statistically significant at the coronal 10 mm level. In a study comparing the removal of biofilm-mimicking hydrogel from artificial isthmuses, the Er: YAG laser demonstrated better results than EDDY [21]. In contrast, our study found EDDY to be superior to the other groups. This discrepancy may be attributed to our use of a diode laser instead of an Er: YAG laser. Additionally, since our study focused on evaluating curved areas, the flexible tip of the EDDY device may have allowed for better access to these regions. The use of polyamide tips, which are much more flexible than rigid metal tips, prevents damage on the canal walls, allows these flexible tips to be advanced to the working length, and according to the manufacturer, EDDY tips move in a high-amplitude three-dimensional manner [12]. According to another study assessing the removal efficiency of calcium hydroxide, this characteristic permits the free transmission of high-frequency oscillations without harming dentin, resulting in rapid fluid movement through three-dimensional oscillation in the coronal third, which may be the cause of its higher efficiency [22]. However, to the best of our knowledge, no previous studies have directly compared these two methods in retreatment in curved root canals, which represent a more realistic clinical challenge. This gap in the literature formed the basis for the present study.

With the introduction of various types of lasers in the field of endodontics, diode lasers have established themselves as reliable tools for root canal cleaning. Additionally, they are capable of eliminating resin sealer remnants and gutta-percha materials during root canal retreatment [11]. Their effectiveness is further enhanced by the creation and quick collapse of vapor bubbles, which produce pressure waves in the irrigating solution. This mechanism facilitates material debridement through cavitation a characteristic that has been particularly noted in diode laser systems operating at near-infrared wavelengths of 810–1064 nm [23]. A study reported that diode laser-assisted retreatment procedures achieved significantly improved root canal cleanliness compared to conventional methods [24]. In another study, Almohareb et al. [11] compared residual gutta-percha remnants in teeth undergoing retreatment and found no significant difference between diode laser and ultrasonic activation in the apical, middle, and coronal thirds. However, both methods were found to be more effective than needle irrigation. In contrast to these findings, although the diode laser yielded better results than needle irrigation in the apical, middle, and coronal thirds in our study, the differences were not statistically significant. This discrepancy may be attributed to the fact that our study was conducted on curved canals, where the diode laser may not achieve optimal effectiveness in the curved regions. Canal geometry affects the distribution and penetration of laser energy [25]. According to earlier research, laser performance in curved root canals may be lower than in straight canals [26, 27]. In a study that measured the laser power transmitted in the coronal, middle, and apical thirds of the root during vertical and horizontal laser applications, it was found that the transmission was lower in the horizontal application, particularly in the apical and middle thirds, than in the coronal region [28]. This leads to the conclusion that the fiber tip will remain closer to the inner wall and further away from the outer wall, this directed energy distribution may cause uneven irradiation of canal walls, where certain areas may receive less energy, potentially reducing the effectiveness of irrigant activation. To the best of our knowledge, no studies have been conducted on the retreatment of curved root canals using diode lasers in the existing literature.

The effectiveness of irrigation activation devices may not always be the same across all regions of the root canal system. As demonstrated in this study, dividing the canal into apical, middle, and coronal thirds allows for a more detailed assessment, focusing particularly on the apical third, which exhibits the greatest curvature and is the most resistant to effective debridement. Consistent with our observations, previous studies have similarly determined that the apical third is the region where persistent residual material is most frequently found [14, 29, 30]. Unlike our study, Lyngdoh et al. did not observe significant differences between the canal thirds within each group. This difference in findings may be attributed to the use of straight canals in their methodology, whereas our study used curved root canals, which are known to present greater challenges in terms of cleaning efficiency [14]. In the present study, the highest amount of residual filling material was observed in the apical 2 mm of the non-activated control group. This finding suggests that, particularly in retreatment cases, even when the coronal region appears adequately cleaned without activation, substantial remnants may remain in the apical portion, which could negatively influence treatment outcomes.

A limitation of the present study can be noted as follows: resin blocks does not accurately mimic the physical properties of natural dentin. In particular, the straight canal configuration, uniformly round cross-sectional shape, smooth canal walls, and lack of a smear layer contrast with the intricacy of actual anatomy. The relative smoothness and flat walls of the resin canals, compared with the irregularities and anatomical complexities of real dentin, may have influenced the removal of obturation remnants. As a result, care should be taken when extrapolating these results to clinical settings with more intricate root canal structures [31]. To the best of our knowledge, no study has evaluated the thermal effects of diode laser activation in resin blocks. However, previous studies conducted on extracted human teeth have shown that diode lasers used at different power settings, modes, and irradiation times result in temperature increases that remain within clinically acceptable limits when appropriate parameters are selected. In the present study, no visible melting or deformation was observed in the resin blocks following laser application [32]. Despite these limitations, transparent resin blocks were used to ensure a high degree of standardization across all specimens. This was essential for a reliable and reproducible assessment of cleaning performance within the curved regions of the root canal system. This approach is consistent with previous investigations that have used resin blocks to maintain uniformity in experiments evaluating irrigation activation and cleaning efficacy. A primary advantage of this model is the ability to provide identical root canal shapes, lengths, tapers, and apical diameters in each sample, effectively excluding confounding factors such as dentin composition or the inherent morphological inconsistencies of natural teeth. Particularly in studies involving curved canals, which are notoriously difficult to standardize in human teeth, as it allows the effect of the irrigation protocol itself to be isolated. Furthermore, the transparency of the material enabled for a clear lateral view during the evaluation process [33–36].

Another limitation of this study is that, although a significant difference was observed between the 2 mm and 6 mm levels in the EDDY group, the comparison between 2 mm and 10 mm showed a numerically considerable difference that did not reach statistical significance. This is likely related to the sample size, and repeating such studies with a larger number of specimens would provide a more substantial contribution to the literature.

Additionally, since all pixel-based measurements were performed by a single examiner in a single session, formal intra-examiner reliability could not be assessed. Future studies should incorporate duplicate measurements at different time points and report intraclass correlation coefficients to further support the reproducibility of image-based analyses.

Considering all of this, even though Eddy has been found to be more effective, particularly in curved areas, more research is required, especially studies that conducted on real tooth, the use of solvents, which is a crucial step in retreatment, and studies using more reliable imaging techniques like micro-CT.

## Conclusions

Within the limitations of the present study, no irrigation activation method has been entirely effective in eliminating filling material remnants from root canals. Among the methods tested, EDDY achieved the best results in the apical 2 mm and middle 6 mm of curved canals, which represents the most challenging region to clean.

## Data Availability

The datasets used and/or analysed during the current study are available from the corresponding author on reasonable request.

## Abbreviations

ANOVA: Analysis of Variance
DL: Diode laser
EDDY: EDDY activation system sonic tip used with an air scaler
EDTA: Ethylenediaminetetraacetic acid
Er:YAG: Erbium-doped Yttrium Aluminum Garnet
ETRB: Endo Training Resin Blocks
Hz: Hertz
mL: Milliliter
mm: Milimeter
μm: Micrometer
NaOCl: Sodium hypochlorite
NI: Needle irrigation
nm: Nanometer
p: Statistical significance, p value
SPSS: Statistical Package for the Social Sciences
